# Gene Expression Profiles of the Immuno-Transcriptome in Equine Asthma

**DOI:** 10.3390/ani13010004

**Published:** 2022-12-20

**Authors:** Elisa Padoan, Serena Ferraresso, Sara Pegolo, Carlo Barnini, Massimo Castagnaro, Luca Bargelloni

**Affiliations:** 1 Department of Comparative Biomedicine and Food Science, University of Padova, 35020 Legnaro, Italy; 2 Department of Agronomy, Food, Natural Resources, Animals and Environment, University of Padova, 35020 Legnaro, Italy; 3 Equine Hospital Padova, 35010 Limena, Italy

**Keywords:** equine asthma, mild equine asthma, MEA, severe equine asthma, SEA, bronchoalveolar lavage, BAL, gene expression, microarray

## Abstract

**Simple Summary:**

The clinical importance and the great economic impact of equine asthma (EA), classified as mild (MEA) and severe (SEA), have led to several studies to better understand its features; however, the fragmented information available does not allow researchers to fully describe the pathophysiology of this disease. Transcriptomic analysis can help in understanding the biological mechanisms underlying equine asthma. To date, the great majority of gene expression studies on equine airway diseases have focused on the analysis of only a few candidate genes, hampering robust conclusions about EA pathogenesis. In the present study, the employment of an immune-specific horse array on SEA and MEA-affected horses helped to comprehensively define their transcriptomic fingerprints. For both diseases, findings suggest that the increased amount of mucus in the airways is due to mucociliary clearance reduction rather than mucus hypersecretion. In addition, excessive complement activation might be responsible for tissue injury. Other biological pathways, mainly those involved in the perpetuation of chronic inflammation and tissue remodeling similar to human asthma, are characteristic of SEA.

**Abstract:**

Background: Mild equine asthma (MEA) and severe equine asthma (SEA) are two of the most frequent equine airway inflammatory diseases, but knowledge about their pathogenesis is limited. The goal of this study was to investigate gene expression differences in the respiratory tract of MEA- and SEA-affected horses and their relationship with clinical signs. Methods: Clinical examination and endoscopy were performed in 8 SEA- and 10 MEA-affected horses and 7 healthy controls. Cytological and microbiological analyses of bronchoalveolar lavage (BAL) fluid were performed. Gene expression profiling of BAL fluid was performed by means of a custom oligo-DNA microarray. Results: In both MEA and SEA, genes involved in the genesis, length, and motility of respiratory epithelium cilia were downregulated. In MEA, a significant overexpression for genes encoding inflammatory mediators was observed. In SEA, transcripts involved in bronchoconstriction, apoptosis, and hypoxia pathways were significantly upregulated, while genes involved in the formation of the protective muco-protein film were underexpressed. The SEA group also showed enrichment of gene networks activated during human asthma. Conclusions: The present study provides new insight into equine asthma pathogenesis, representing the first step in transcriptomic analysis to improve diagnostic and therapeutic approaches for this respiratory disease.

## 1. Introduction

In 2017, the term “equine asthma” was proposed to unify the noninfectious inflammatory lower airway diseases of horses, such as inflammatory airway disease (IAD), recurrent airway obstruction (RAO), and summer pasture-associated obstructive airway disease [1]. Inflammatory airway disease (IAD), now called mild equine asthma (MEA), and recurrent airway obstruction (RAO), called severe equine asthma (SEA), are two of the most frequent equine airway inflammatory diseases.

MEA is an inflammatory disease mainly affecting young horses up to 6–8 years of age that shows poor performance and is generally associated with abnormal breathing during exercise, nasal discharge, and coughing. According to the latest consensus statement [2], inflammation can be characterized by eosinophilic, neutrophilic [3], or mast cell infiltration [4] in the airways. Stabling (i.e., bedding material), which could promote the presence of allergenic dust or microbiological contaminants, and contact with horses affected with infectious airway diseases represent two important predisposing factors for the development of MEA [5,6,7]. The clinical importance and the great economic impact of this disease led several authors to study its clinical, endoscopic, and cytological parameters to better understand its features (i.e., etiologic agent and pathological mechanisms) [8,9,10]. Only recently have the expression profiles of some immune-related genes in the broncho-alveolar lavage (BAL)-fluid obtained from MEA-affected horses been evaluated [4,11,12]. However, the results are highly controversial, as the expression profiles of the commonly investigated transcripts are often not consistent among the various studies. These differences could be due to many factors, including the fact that the sampling was performed in different stages of the disease, the lack of cellular differentiation of the airway inflammation, the absence of microbiological examination, or specific individual variations. Previous studies showed the presence of either mast cells, neutrophils, or eosinophils in the BAL of MEA-affected horses, suggesting the involvement of various inflammatory mediators in relation to the phenotype. Beekman et al. [4] observed differences in the gene expression profiles of IL-17 and IL-4 between MEA-affected horses characterized by a mast cell infiltrate and those having a neutrophilic infiltrate.

SEA is a chronic inflammatory airway disease affecting mainly adult horses. It is characterized by both genetic predisposition and the involvement of environmental factors [13]. SEA-affected horses develop bronchoconstriction, airway neutrophilic inflammation, hypersensitivity of the respiratory tract associated with mucus hypersecretion, and, in the most severe cases, tracheal and bronchial edema. Clinically, SEA-affected horses show nasal discharge, increased respiratory rate at rest, chronic cough, and exercise intolerance.

Chronic airway inflammation and obstruction, typical of SEA, have many similarities with the changes in the respiratory tract in human asthma, suggesting the potential activation of common immunological mechanisms [14,15,16]. Although some studies [17] showed an increase in Th1 cytokine mRNA levels, others [18] reported activation of the Th2 response characterized by IL-4 and IL-13 overexpression in BAL cells. As presumed also for MEA, these contrasting results could be determined either by the different individual responses to the disease or by sampling times [19]. Recently, Padoan et al. [20] reported the upregulation of IL-1β, IL-8, TGF-β1, NF-kβ, TLR4, and TNFα in the BAL fluid of SEA-affected horses compared with healthy controls. Therefore, the inflammatory component of SEA appears to result from a contribution of both the innate and the adaptive immune response, with the consequent development of type I and type III hypersensitivity reactions accompanied by an increased expression of both Th1 and Th2 cytokines.

However, the fragmented information available does not allow us to accurately define the inflammatory response in both SEA and MEA. Therefore, a broader view is needed to better depict the immunological mechanisms involved in the development of both acute and chronic airway inflammation.

Advances in biotechnological research have led to the development and application of more comprehensive tools based on “transcriptome fingerprinting” to obtain a deeper understanding of the disease’s mechanisms, especially in humans [21,22,23]. In the context of human lung diseases, Woodruff and colleagues [24] applied DNA microarray technology to compare the gene expression profiles of bronchial epithelial brushings from asthmatic patients, showing the inhibitory effect of corticosteroids on some genes expressed in the bronchial tree. Microarray analysis was also applied to animal models (mice) of chronic lung disease with pathological findings that resemble those observed in human asthma and chronic obstructive pulmonary disease (COPD) [25].

To date, this technology has been applied only to a limited extent to study the veterinary counterpart of pulmonary diseases. A pioneering study by Ramery and colleagues [26] compared the gene expression profiles in BAL cells and in the peripheral blood cells of the SEA-affected horse versus controls using a human gene chip. However, the results showed the obvious limitations of applying a human platform to another animal species, as only 3 upregulated and 10 downregulated genes in the BAL cells of SEA-affected horses were identified. More recently, Korn and colleagues [27] evaluated the gene expression profiles in the mediastinal lymph nodes of SEA-affected horses by developing a custom microarray platform. Thanks to this approach, a higher number of differentially expressed genes in SEA-affected horses was reported, supporting an IL-17 response.

The use of microarray technology could therefore allow deeper investigation into the molecular mechanisms of innate and acquired immune responses that are involved in the development of inflammatory airway diseases in horses.

In the present study, an equine DNA microarray platform was developed by selecting approximately 7000 immune-related genes to study the expression profiles of BAL from SEA- and MEA-affected horses. The results obtained were also correlated with clinical, endoscopic, cytological, and microbiological findings. The aim was to explore in more detail the immunological mechanisms underlying the development of mild and severe equine asthma and to assess the potential correlations of the gene expression profiles with the investigations conducted daily in clinical practice.

## 2. Material and Methods

### 2.1. Animals

A total of 25 horses were examined for this study, including 7 clinically healthy standardbred horses age 6 ± 1 years, 3 males and 4 females (CTRL group); 8 SEA-affected horses, 4 males and 4 females (2 standardbred and 6 saddle horses) age 12 ± 1 years old (SEA group); and 10 MEA-affected horses, 5 males and 5 females (3 standardbred and 7 saddle horses) age 6 ± 1 years old (MEA group). The horses were assigned to one of the three groups after the determination of their clinical status. For SEA- and MEA-affected horses, sampling was performed at the time of the clinical case presentation.

BAL specimens were obtained from cases that presented to the Equine Patavium Hospital (Padova, Italy) as part of routine diagnostic examination. For healthy horses, BAL fluid was collected from animals voluntarily enrolled by their owners to obtain a complete examination of the free airways. Informed consent was obtained from all owners. The research did not involve animal experimentation or euthanasia.

### 2.2. Clinical Examination

All horses included in the study were examined by the same clinician [10]. The clinical status was determined by considering parameters including heart rate, respiratory rate, and lung sounds. Endoscopy of the upper and lower respiratory tract was performed after sedation (xylazine 1.1 mg/kg). A clinical score was assigned to each animal similarly to previous studies [28,29]. The horses exhibiting cough, nasal discharge, increased respiratory rate, nostril flaring, abdominal effort at rest, wheezing, mucus hypersecretion, and hyperemia/edema of the respiratory tract associated with a past history of chronic respiratory disease were included in the SEA group. The horses with a history of poor performance, abnormal lung sounds appreciated with the rebreathing bag, mucus hypersecretion on endoscopic examination, and neutrophilic inflammation at cytological analysis were included in the MEA group. The study was performed only on MEA-affected horses characterized by neutrophilic inflammation that did not have mast cells or eosinophilic inflammation of the lower respiratory tract. During endoscopy, each respiratory condition of the animals was assigned a score for the degree of hyperemia and mucus hypersecretion [30].

The horses included in the control group showed no relevant clinical signs amenable to respiratory or other diseases or a history of poor performance. Moreover, the CTRL horses did not show any pathological changes in the respiratory tract during endoscopic examination.

### 2.3. Bronchoalveolar Lavage (BAL) Examination

Bronchoalveolar lavage (BAL) was conducted by introducing 180 mL of sterile isotonic saline solution and 60 mL of sterile lidocaine solution 2% through the sterile accessory part of the endoscope in both primary bronchi. The fluid retrieved was filtered through sterile gauze and divided into three aliquots for bacteriological and cytological examination and gene expression analysis. The first aliquot was centrifuged at 2500 rpm for 10 min. The sediment obtained was soaked in one ml of RNAlater^®^ (Ambion, Life Technologies, Carlsbad, CA, USA), kept for one night at +4 °C, and then stored at −20 °C until total RNA extraction. In order to exclude bacterial infection of the lower airways, bacteriological examination was performed on the BAL fluid of all animals. For bacteriological examination, McConkey agar and the agar blood media were incubated at 37 °C ± 1 °C for 24 h with 20 μL of the BAL fluid in each. In addition, 100 μL of BAL fluid was added to an enrichment broth and incubated at 37 ± 1 °C for 24 h. The bacterial colonies were subsequently transferred to blood agar and incubated at 37 ± 1 °C for 24–48 h. For cytological examination, a smear of the fluid and one of the pellets, which resulted from centrifugation of 5 mL of BAL fluid at 1500 rpm for 10 min, was made. All slides were stained with May-Grünwald Giemsa. In addition, a differential cell count was performed by counting a total of 200 cells per smear on the stained slides.

Total RNA extraction from BAL cells was performed as previously described [20]. For each sample, RNA concentration was measured using a UV–Vis spectrophotometer NanoDrop ND-1000 (Nanodrop Technologies, Wilmington, DE, USA), and RNA integrity was determined by running each sample on an RNA-chip in an Agilent 2100 Bioanalyzer (Agilent Technologies, Palo Alto, CA, USA). To reduce experimental biases caused by poor RNA quality, only samples characterized by an RIN ≥6.5 were included in the microarray analysis.

### 2.4. Gene Selection and Microarray Platform Design

**Figure 1:**
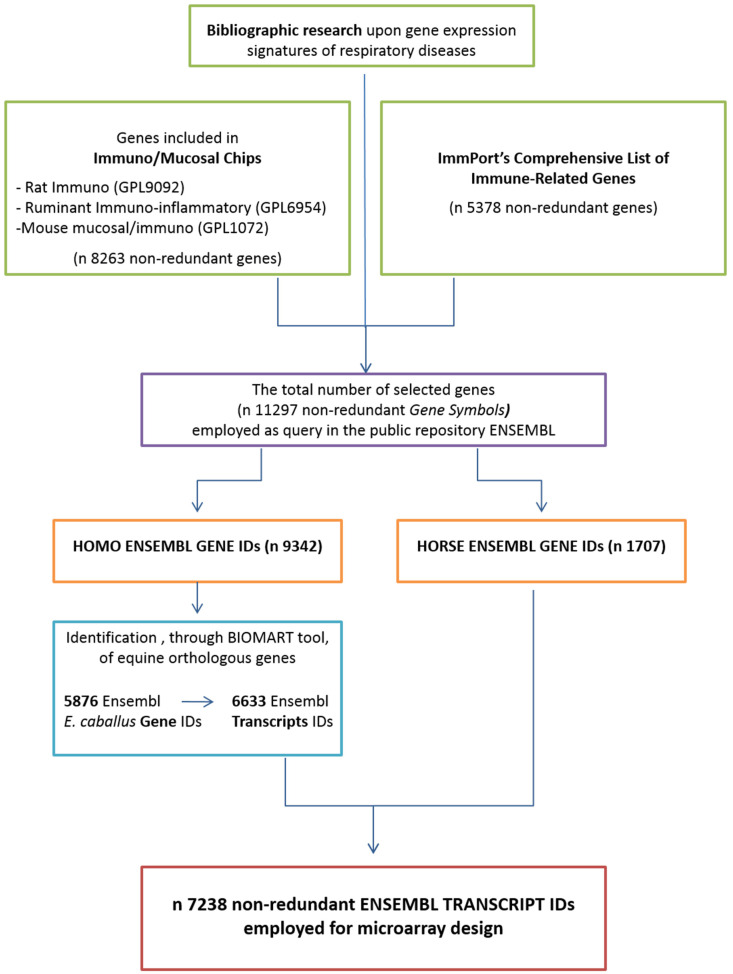
**Microarray design.** Schematic diagram of the approach used for the selection of E. caballus transcripts included in the microarray platform.

The process of gene selection for the microarray platform design focused on the immune system and mucosal genes, as described in Figure 1. Briefly, three different approaches were integrated: (i) genes included in 3 immune and mucosal chips deposited in the public database GEO (Gene Expression Omnibus) were selected: Rat Immune Microarray (GPL9092), Ruminant Immuno-inflammatory array (GPL6954), and Mouse mucosal/immuno (GPL1072); (ii) ImmPort’s list (comprehensive list of immune-related genes, 5378 nonredundant genes) of the genes involved in the human immune response was collected; and (iii) the genes studied in equine and human respiratory diseases in recent years by several authors [31,32,33,34,35] were listed. These approaches led to the selection of 11,279 human genes for which *E. caballus* orthologs were retrieved by using the Ensembl BioMart data mining tool. In the end, a total of 7238 Ensembl equine Transcript IDs were used for microarray probe design. The probes (60-mers) were designed using the software eArray (Agilent Technologies, Palo Alto, CA, USA) and synthesized in duplicate through SurePrint (Agilent Technologies, Palo Alto, CA, USA) technology to obtain an 8 × 15 k microarray format.

### 2.5. Microarray Data Acquisition

Sample labeling and hybridization were performed as reported by Ferraresso et al. [36]. Hybridized slides were scanned at a 5 μm resolution using an Agilent G2565BA DNA microarray scanner. Default settings were modified to scan the same slide twice at two different sensitivity levels (XDR Hi 100% and XDR Lo 10%). The images generated from the scanner for each slide were analyzed using Agilent Feature Extraction 9.5.1 software. For each array, the software provides a series of quality measures in order to evaluate the goodness and the reliability of spot intensity estimates, including the estimation of the normal distribution of fluorescence values.

### 2.6. Statistical Analysis

Fluorescence data for all samples (CTRL, MEA, and SEA) were normalized together with a cyclic loess approach to avoid the introduction of bias. This approach was chosen because after normalization the spike intensity should be uniform among the different experiments of a given dataset. After further quality control, based on the exclusion of the probes characterized by an intensity value lower than the lowest concentration of the spike-in, the probes characterized by an intensity value < 4.5 (close to the limit of detection of the method) in at least 19 samples (75%) were excluded from the statistical analysis. Filtering and normalization were performed using the statistical software R. Principal component analysis (PCA) of normalized and filtered gene expression data was performed using the TMEV suite. To identify the differentially expressed genes between the CTRL group and the MEA- or SEA-affected horses, a two-class nonparametric test for unpaired data was implemented in the program Significant Analysis of Microarrays (SAM). The false discovery rate (FDR) was set to 0.1, and the fold-change (FC) was set to 1.5.

Gene Set Enrichment Analysis (GSEA) was performed on all microarray expression data to identify potential pathways enriched in MEA and SEA without differential cutoff selection. First, gene sets available in the Molecular Signature Database (MSigDB, www.gsea-msigdb.org, accessed on 30 April 2019) that are implicated in the immune response were selected (C2: curated gene sets, Canonical Pathways). Next, new gene sets were created using transcripts commonly investigated in both human and equine respiratory diseases. Finally, the gene sets enriched in human respiratory diseases submitted by Chowdhary and colleagues [37] were also included to evaluate a potential enrichment of common pathways in equine and human respiratory diseases [38].

The Signal2Noise metric for ranking genes was applied, and gene sets were considered significantly enriched when the false discovery rate (FDR) was below 0.25, according to GSEA recommendations.

Spearman’s nonparametric statistical test was performed using the commercially available IBM statistical software SPSS, version 20 (SPSS v20.0, IBM, Armonk, NY, USA) to evaluate potential correlations between the percentage of exfoliated epithelial cells detected in the cytological examination and the expression values of genes affecting the behavior of the ciliated epithelial cells in the respiratory tract. A *p* value < 0.05 was considered significant.

## 3. Results

### 3.1. Clinical Examination

Clinically, a significant difference in respiratory rate at rest between SEA- and MEA-affected horses was appreciated, and it was significant in the t test analysis (*p* = 0.009). In addition, the amount of mucus detected during endoscopic examination in the tracheal lumen and in the explored bronchi was significantly different between the SEA and MEA groups (mean mucus hypersecretion 3 ± 0.7 and 2 ± 0.9, respectively; *p* = 0.03).

The cytological analysis of BAL fluid was performed by evaluation of the inflammation pattern, identification of cellular alterations, and assessment of the presence of microorganisms. Bacteriological examination was negative for all control and MEA- and SEA-affected horses. The results of the differential inflammatory and epithelial cell counts are reported in Table 1.

Already reported as being the first line of defense in the airways of all species, equine included [39], macrophages represented more than 95% of counted cells in CTRL horses. In MEA-affected horses, macrophages were still the prevailing cell type, although a neutrophilic infiltrate also characterized the airways of the MEA-affected horses, while SEA-affected horses showed prevalent neutrophilic airway inflammation.

### 3.2. Microarray Data Analyses

**Figure 2:**
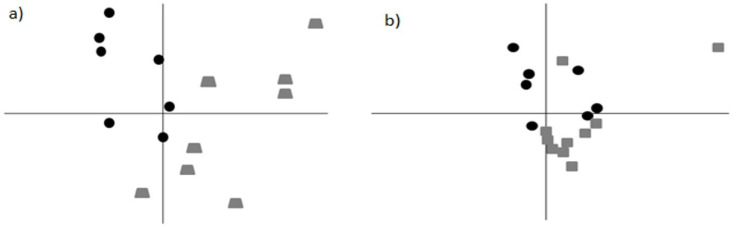
**PCA of gene expression data.** (**a**) Comparison between the control group (black circles) and SEA-affected horses (gray trapezoids). The **x**-axis and **y**-axis represent 55% and 11% of the total variance, respectively. (**b**) Comparison between the control group (black circles) and MEA-affected horses (gray squares). The total variance was represented by 40% of the **x**-axis and by 21% of the **y**-axis.

After data extraction, normalization, and filtering, processed signals for 6045 unique transcripts were analyzed in 25 BAL samples using PCA as an unsupervised method to identify differences in gene expression profiles of MEA- and SEA-affected horses compared with controls (Figure 2).

PCA confirmed a separation between the groups of horses previously distinguished by clinical assessment and by endoscopic and cytological examination (CTRL, SEA, and MEA groups). The first two components, which explained a considerable fraction (66%) of the total variance, clearly separated the controls from the SEA-affected horses (Figure 2a). A less pronounced separation was instead observed between the control and MEA-affected horses (first two components = 61% total variance) (Figure 2b). In both cases, no clustering according to sample sex was found.

To explore the statistical significance of the observed separation, a two-class unpaired SAM test was performed to identify differentially expressed genes between controls and pathological samples. Using an FDR of 10% and an FC of 1.5, a total of 1763 differentially expressed genes (DEGs, 903 overexpressed and 860 underexpressed) were found between the SEA and CTRL groups, with fold-changes ranging from 15.8 to −13 (Table S1).

Many upregulated genes play roles in promoting and sustaining hematopoietic cell differentiation (e.g., M-CSF, TREM2, IL1A, LCP2, and VAV1), NF kappa B signaling (e.g., RELB, NFKB1, NFKB2, and BCL2), and the inflammatory response (e.g., TREM1, VCAM1, IL4R, CCL17, IL1A, IL1B, and IRAK2). It is noteworthy that several downregulated genes are involved in epithelial tight junction composition (e.g., CLDN7, CLDN8, TJP2, TJP3, and PARD6B) and either the synthesis or O-glycosylation of mucins (e.g., MUC1, MUC4, MUC5AC, and GALNT10). While both pathways play central roles in airway defense, their dysregulation results in defective lung protection via either epithelial barrier breakdown or compromised ciliary clearance.

The comparison between MEA and CTRL groups yielded a lower number of DEGs with lower fold-changes (ranging from 2.8 to −5.4), thus reflecting the less evident separation from CTRLs observed in the PCA. SAM analysis identified 379 DEGs (55 overexpressed and 324 underexpressed; see Table S2). Among the upregulated transcripts, several genes involved in the regulation of apoptosis (e.g., CASP10 and TNFRSF10A) and RUNX3-mediated transcription (e.g., RUNX3 and RUNX1) were observed. Upregulated genes were also found to participate in tight junction organization (e.g., ACTN1, VASP, MSN, and ARHGEF2). Of particular interest is the downregulation of many genes implicated in cilium assembly (e.g., NPHP1, IFT27, IFT88, IFT57, TUBA1A, NPHP4, and DYNLL1) as well as complement coagulation cascades (e.g., CFI, CD46, F3, PLAT, and C3).

**Figure 3:**
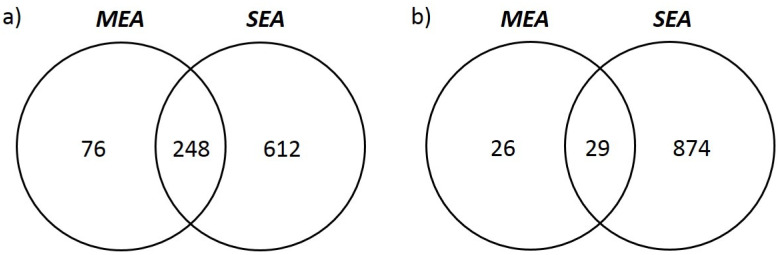
**Common DE genes between MEA- and SEA-affected horses.** Venn diagrams showing the number of common differentially expressed genes between MEA- and SEA-affected horses. MEA: pairwise comparison between the MEA and CTRL groups. SEA: pairwise comparison between the SEA and CTRL groups. (**a**) Upregulated transcripts; (**b**) downregulated transcripts.

The comparison between SEA and MEA DEGs revealed 248 downregulated and 29 upregulated genes that are shared in both diseases, showing a highly significant concordance (Fisher’s exact test, *p* < 0.0001; Figure 3). Functional annotation of commonly upregulated genes provided no significant result, while seven pathways were found to be significantly enriched when considering commonly downregulated genes. These pathways are largely redundant and can be traced back to “cilium assembly” (q-value 1.38 × 10^−7^) and “complement and coagulation cascades” (q-value 0.0061).

A two-class unpaired SAM test was also performed to investigate differentially expressed genes between SEA and MEA samples. Even if the PCA showed a separation between the two groups, the number of DEGs was considerably lower compared with those found against CTRLs. In total, 20 and 3 genes were found to be upregulated and downregulated in the SEA group, respectively (see Table S3), with upregulated genes in SEA mainly involved in regulating the inflammatory response (e.g., RELT, CD1c, CD1E, and SLAMF9).

### 3.3. Gene Set Enrichment Analysis (GSEA)

Gene set enrichment analysis was performed on 75 gene sets. Selected gene sets included immune-related pathways available in the MSigDB (see Methods) together with gene sets related to human respiratory diseases reported by Chowdhary et al. [34]. For both the MEA and SEA groups, the whole experimental dataset (normalized expression data of 6045 genes) was employed in comparison to the CTRL group. In the comparison between the MEA and CTRL groups, no gene set was found to be significantly enriched with an FDR q value ≤ 0.25. When comparing the SEA and CTRL groups, 38 gene sets were significantly enriched with FDR q value ≤ 0.25, with a total of 18 gene sets with FDR q value ≤ 0.25 and nominal *p* value ≤ 0.05 (see Table 2). Gene sets related to activation of inflammation (i.e., BIOCARTA_STRESS_PATHWAY, BIOCARTA_IL7_PATHWAY, KEGG_CYTOKINE_CYTOKINE_RECEPTOR_INTERACTION, and KEGG_LEUKOCYTE_TRANSENDOTHELIAL_MIGRATION) were among the most significantly enriched. Notably, the gene sets identified by Chowdhary et al. [37] as being associated with human COPD, emphysema, and asthma were all significant, the latter at nominal *p* value ≤ 0.05 (nominal *p* value: 0.048, FDR q value: 0.11).

## 4. Discussion

Several authors have investigated the immunological mechanisms underlying the immune response in mild and severe equine asthma. However, knowledge about its pathogenesis is still limited and fragmented. Information about the expression profiles of few candidate genes often does not allow robust conclusions about the pathogenesis and the involvement of innate and/or adaptive immune responses.

In the present work, we developed a horse-specific transcriptomic platform aiming at obtaining a more comprehensive picture of this disease to contribute to an improvement of diagnostic and therapeutic approaches. All genes found to be differentially expressed, and the corresponding fold changes are listed in Table S1 and Table S2 for the SEA and MEA groups, respectively.

### 4.1. MEA

A significant number of transcripts overexpressed in MEA are directly involved in the inflammatory response, particularly in the IFN-activated pathway (i.e., FI44 L and SIGLEC1), LPS recognition by macrophages (MSN), and prostaglandin biosynthesis (PTGS2).

The activation of type I IFN-dependent pathways is a consequent response to viral and other microbial infections, and it also has an immunomodulatory effect on activating natural killer (NK) cells, macrophages, and dendritic cells (DCs), all of which are essential effectors of the innate immune system. By virtue of their effect on DC maturation, type I IFNs are currently recognized as pivotal cytokines bridging two aspects of host defense: innate and adaptive immune systems [40]. MSN functions as an independent receptor on human monocytes for the bacterial endotoxin LPS and appears to play a role in the transduction of all LPS-induced signals, including the proinflammatory activities mediated, at least in part, by CD14 [41]. PTGS2, better known as cyclooxygenase 2 (COX2), is primarily an inducible isoform of the key enzyme for prostaglandin biosynthesis, and its expression can be upregulated in many cell types by cytokines, mitogens, and endotoxins. PTGS2 is highly expressed in inflamed tissues and is believed to produce prostaglandins involved in inflammatory processes [42]. Finally, TNFRSF10A (also known as DR4 or TRAILR-1) was found to be overexpressed in MEA-affected horses. TRAIL is a member of the TNF superfamily and acts as a type II membrane protein to induce apoptosis in a variety of target cells. In some cell types, TRAIL binding to the death receptors DR4, DR5, and/or DcR2 may also activate the transcription factor NF-κB, leading to the transcription of genes that actually antagonize death signaling pathways and promote inflammation [43]. In addition, neutrophils overexpress TRAIL in response to certain proinflammatory cytokines [44]. While all of the above evidence points to the activation of proinflammatory pathways, the upregulation of IL10RA, which is part of the cytokine IL-10, also suggests the presence of signals to counterbalance the inflammatory reaction. IL-10 inhibits the synthesis of INF-γ by T lymphocytes, promoting the development of a Th2 cytokine profile. IL-10 also inhibits proinflammatory mediators, including PTGS2 [45]. Overexpression of IL10RA is in agreement with evidence from Beeckman et al. [4], who reported the upregulation of IL-10 in BAL cells of horses with MEA.

In the present work, we decided to include only samples characterized by a neutrophilic infiltrate. However, although the obtained results indicate the activation of proinflammatory pathways, it is still difficult to determine whether a predominant Th1 or Th2 immune response has developed.

### 4.2. SEA

SEA has been suggested to be the pulmonary disease naturally occurring in animals that is most similar to human asthma. In the present study, analysis of the SEA transcriptome highlighted some interesting results. Chemokine CC Motif Ligand 17 (CCL17) was found to be the most upregulated transcript (FC 15.8). This chemokine is released by M2 macrophages and activated by the proinflammatory mediators IL-4 or IL-13, and it acts by promoting the activation and migration of T lymphocytes to the site of inflammation. Staples and colleagues [46] reported the overexpression of CCL17 in human asthmatic patients, suggesting the involvement of the PI3-Kinase (PI3K) pathway in IL-4-induced CCL17 production.

Notably, in the present study, both the IL-4 receptor and PIK3CG were upregulated in the SEA group. PI3K is known to promote the activation of the IL-17, IL-2, and TGF1β pathways. The involvement of IL-17 in SEA pathogenesis has been previously suggested by Korn and colleagues [27], who found significantly increased IL-17 immunoreactivity in lymph node sections of SEA-affected horses even if IL-17 mRNA expression itself was not dysregulated. In fact, in our study, the genes encoding IL-17 subunits A-C-F were not differentially expressed. In contrast, TGF1β was overexpressed in SEA. It plays a key role in stimulating the conversion of epithelial cells into fibroblasts and myofibroblasts. This mechanism, associated with collagen overproduction, appears to be involved in bronchial fibrosis in human asthma and COPD [47]. The binding between TGF1β and its receptor may activate a SMAD-dependent signaling pathway and a SMAD-independent one [42]. In vitro, the conversion of human respiratory epithelial cells (and in particular bronchial epithelial cells) into mesenchymal tissue stimulated by TGF1β appears to follow the SMAD-dependent pathway, which includes the activation of SMAD3 and SMAD4 [48,49]. In the present study, no significant differences in the expression levels of SMAD3 and SMAD4 between the SEA and CTRL groups were found, while overexpression of TRAF6 and TAK1, the activation of which promotes the SMAD-independent pathway, was observed. The hypothesis of a predominant involvement of the SMAD-independent pathway is also supported by overexpression of SMAD7, which acts as an inhibitory molecule of the SMAD-dependent pathway [50,51].

A similarity with the mechanism of bronchoconstriction, which characterizes airway remodeling in human asthmatic patients, appears to be confirmed by overexpression of MMP-9 and TIMP-1 [52,53]. MMP-9 expression and gelatinolytic activity were recently found to be significantly increased in BAL of horses with asthma (i.e., RDLAI, Lower Airway Inflammation with Respiratory Distress, [54]) and it is suggested that they are involved in the MMP-dependent proliferation of smooth muscle cells, a structural change that appears in asthmatic airways. Another study [55] reported increased levels of metalloproteinases and their inhibitors, in particular MMP-9 and TIMP-1, in equine chronic pneumopathies including RAO, suggesting these molecules have prognostic value. The same authors also hypothesized that in equine asthma, overexpression of MMPs contributes to pathological tissue destruction, while TIMPs counteract MMPs with overexpression, leading to fibrosis formation [56].

Moreover, considerable overexpression of INHBA in SEA-affected horses might also suggest a role for this molecule in the bronchoconstriction process, as it was proven to stimulate bronchial cells’ proliferation and extracellular matrix remodeling [57].

Dysregulation of apoptosis has been implicated in allergic asthma and COPD in humans. Breuer et al. [58] and Moran et al. [59] conducted studies on the apoptotic response of different lymphocyte subpopulations in SEA-affected horses. They showed that delayed apoptosis could promote persistent inflammation and related tissue damage. Neutrophil/macrophage apoptosis was also proven to be altered in the BAL fluid of SEA-affected horses compared with healthy animals, with a significantly higher percentage of viable neutrophils in SEA [60]. In the present study, overexpression of genes involved in both promoting and inhibiting apoptotic mechanisms, such as FAS, CASP8, CFLAR, MAPK8, and PDCD1, was observed in the SEA group. Notably, CSF1, which plays a key role in granulocyte survival, was found to be strongly upregulated in the SEA group (FC 5.7). The anti-apoptotic effect of CSF1 in heaves-affected horses has been proven to be mediated by STAT5, which, in turn, induces the transcription of BCL2L1 [60]. In the present study, both STAT5 and BCL2L1 were overexpressed in the SEA group, suggesting that STAT5 activation by CSF1 may be involved in delaying granulocyte apoptosis [61].

The immunological processes responsible for the persistent airway inflammation in SEA horses are still largely unknown, although a role of HIF1 has been hypothesized. HIF1 is a major transcription factor responsible for regulating cellular adaptation to hypoxic conditions and is also a master regulator of inflammation. Indeed, it has been proven to regulate many proinflammatory genes, such as TNFα, IL8, VEGFA, and CXCR4 [62,63]. HIF1 activity is dependent on the abundance of the oxygen-regulated HIF1 alpha subunit (HIF1α), which has been reported as being upregulated in alveolar macrophages and lung parenchyma in asthma, and its expression was found to be increased in SEA-affected horses [64].

Unfortunately, at the time of microarray design, the HIF1α gene was not annotated on the *E. caballus* draft genome, making it impossible to investigate its mRNA levels in the present study. However, several HIF1 target genes were found to be upregulated in the SEA group compared with the CTRL group, with FCs ranging from 1.6- to 4-fold (TNFα, CXCR4, IL8, and VEGFA), providing indirect evidence of increased HIF1 activity in SEA-affected horses. In this respect, the strong downregulation of DCN (FC −12.9), which is known to inhibit HIF1 and VEGF expression, is also worth noting [65].

Meanwhile, the significant overexpression of one of the major regulators of HIF1α, FIH1 (factor inhibiting hypoxia, aka HIF1αN), appears to be contrasting evidence. FIH1 is an asparaginyl hydroxylase that is usually upregulated under normoxic conditions and contributes to the inactivation of HIF1α. However, FIHs are HIF target genes themselves, and their upregulation under hypoxia results in a negative feedback loop that reduces HIF1α stabilization [66], providing a possible explanation for this paradoxical observation.

Matrix metalloproteinases (MMPs) might also contribute to SEA pathogenesis. In particular, MMP-1 and MMP-9, both found to be overexpressed compared with CTRLs, have been reported to be associated with COPD severity [67,68], even if their role has not been elucidated. In RAO-affected horses, the positive correlations between MMP-9 expression and neutrophil percentage led to the hypothesis that these cells are the main producers of MMPs [56]. In turn, MMPs might be involved in recruiting inflammatory cells through the airway, thus contributing to sustaining the inflammatory picture typical of SEA disease. This is the case for MMP-9, the levels of which are increased in BAL fluid from patients with asthma, and its role in controlling the T-cell response to allergens and in airway remodeling has been proven [69]. MMP-1 overexpression in the lung has been reported to be associated with emphysema in humans [68]; notably, this gene was also found herein to be the most upregulated in the comparison between SEA and MEA.

Comparing the present findings with those of Ramery and colleagues [70], GADD45A and PTX3 were similarly upregulated. GADD45 expression levels usually increase under stress conditions, including hypoxia, which could lead to cell growth arrest and DNA damage [71]. PTX3 is involved in innate resistance against pathogens, mainly acting at the infection and inflammation sites. Overexpression of this gene may be responsible for increased production of nitric oxide and other proinflammatory molecules as well as for neutrophilic recruitment in the airways of SEA-affected horses [26].

The gene set enrichment analysis highlighted several molecular pathways that were enriched in the SEA group. A significant enrichment of the asthma pathway [72], grouping 174 genes, was found. Among these, IL4R has become relevant, as IL4 is one of the key interleukins involved in the development of this disease. Among the frequent genes, PIK3CG and IRAK3 play a very important role. Some studies in human asthmatic patients demonstrated that PIK3CG inhibition leads to an improvement in respiratory signs, both decreasing Th17 cell differentiation and smooth muscle cell proliferation [25]. These processes result in a reduction in airway inflammation and in the mitigation of bronchoconstriction. This could suggest a possible use of PIK3CG as an inhibitory target to reduce the clinical symptoms in SEA-affected horses. On the other hand, IRAK3 has recently emerged as a susceptibility candidate gene for asthma [73].

Interestingly, the NF-κB pathway was also significantly enriched. Activation of NF-κB appears to be the basis for increased expression of many inflammatory genes and for the perpetuation of chronic airway inflammation in asthma. The synthesis of cytokines, such as TNF-α (upregulated 1.6-fold in the SEA group), IL-1β (upregulated 1.8-fold), IL-6 (IL6 signal transducer was upregulated 2.3-fold), and IL-8 (IL-8 precursor was overexpressed 3.4-fold) is mediated by NF-κB [74].

The involvement of the innate component of immunity through the enrichment of the Toll-like receptor pathway with strong upregulation of TLR4 confirmed previous results [20,38].

From these findings, many similarities with human asthma were observed. In addition, the involvement of both innate and acquired components of immunity was also confirmed, and a prevalent Th2-response seemed to be shown.

### 4.3. Commonly Regulated Genes

Analysis of MEA- and SEA-affected horses revealed hundreds of genes whose expression is increased or decreased in both diseases, suggesting that they might share some biological mechanisms. Functional annotation of commonly upregulated genes produced no significant results, likely due to the low number of shared genes [32], while the analysis of commonly downregulated genes yielded highly significant results. The cilium assembly pathway was the most significantly enriched (q value: 1.4e-07). Modulation of genes regulating length, growth, and motility of cellular cilia [75] was observed in both diseases. In particular, underexpression of RSPH9, IFT57, IFT74, IFT27, IFT81, IFT88, IFT20, and BBS2 was noted in the MEA group when compared to CTRL. Downregulation of these transcripts may suggest reduced respiratory mucociliary clearance in MEA-affected horses. The same genes plus IFT52, IFT122, IFT140, IFT80, IFT172, NEK3, NEK4, NEK11, and BBS4 were underexpressed in SEA-affected horses. In particular, BBS2 and BBS4 are expressed in the airway epithelia, and both BBS2- and BBS4-deficient mice were shown to have abnormal motile cilia structure and reduced beat frequency [76]. The greater number of underexpressed genes affecting ciliary behavior in SEA compared with MEA patients might suggest worse mucociliary clearance in severe asthma than in mild asthma. This hypothesis seemed to be further confirmed by the marked overexpression of ET1 observed in SEA patients. Sabater et al. [77] showed that, apart from being an effective constrictor of bronchial smooth muscle, ET1 significantly depressed the mean tracheal mucus velocity (index of mucociliary clearance) in ovine airways. This finding seems to be supported by the observed increase in the amount of mucus in the SEA group compared with the MEA group. The mucociliary clearance ability in SEA-affected animals is still a controversial issue. Some authors showed, through clinical trials, a decreased clearance ability in horses affected by severe asthma [78], while others did not identify any differences compared with the control group [79]. Mucus hypersecretion is due to complex processes, which include mucin gene transcription, translation into polypeptides, glycosylation of the polypeptide backbones, and goblet cell hyperplasia [80]. Contrary to what has been reported for human COPD, in the present study, marked downregulation of MUC1, MUC4, and MUC5AC was observed in the SEA group. These genes encode for respiratory muco-protein film production [80]; in particular, MUC5AC is predominantly found at the protein level in mucus and sputum.

These findings suggest that the increased mucus amount in the airways of MEA- and SEA-affected horses could be due to a mucociliary clearance reduction rather than mucus hypersecretion. No statistical correlation between the number of epithelial cells found in cytological examination and the expression values of genes influencing the function of the ciliary apparatus was observed. This appears to exclude the hypothesis that the decreased expression of genes coding for cilia behavior could be determined by a larger amount of inflammatory cells compared to the epithelial cells in the BAL fluid of the MEA- and SEA-affected animals.

The other significantly enriched pathway was “complement and coagulation cascades,” with several genes found to be downregulated in both diseases (i.e., CFI, CLU, F3, C3, PLAT, SERPINA1, and CD46) compared with the controls. In particular, CFI, CD46, and PLAT are complementary inhibitors involved either in the inactivation or in preventing the assembly of the complement components; thus, their underexpression might reflect increased activation of the complement cascade, as already reported by Moran et al. [59]. Altered expression of complement regulatory proteins, which lead to excessive complement activation, can contribute to tissue injury. This has been proven in humans, where loss of lung function in patients with COPD and emphysema was associated with a lower expression of CD46 [81]. A comparable role for complement activation might be hypothesized in the case of SEA and MEA. Finally, in both MEA and SEA patients, compared with the control group, downregulation of SERPIN1 was shown. This gene encodes an elastase inhibitor [82,83]. Decreased expression of SERPIN1 in MEA and SEA-affected horses could have an important role in tissue remodeling, which drives peribronchial and peribronchiolar fibrosis.

## 5. Conclusions

To date, the majority of gene expression studies on equine airway diseases have been limited to the analysis of only a few candidate genes, which are usually based on previous knowledge of model species. The employment of an immune-specific horse array for investigating the expression profiles in the BAL fluid of SEA and MEA-affected horses contributed to the advancement of our understanding of the pathogenesis and the immunological mechanisms underlying equine asthma. Several pathways were found to be significantly altered in both diseases, while others, mainly involved in the perpetuation of chronic inflammation and tissue remodeling, are characteristic of SEA. As the number of cases included in the present study is limited, these findings definitely deserve further investigation in a wider cohort to obtain more robust results. An increased number of subjects would also allow us to investigate expression patterns directly correlated with the severity of the disease and to identify candidate targets of therapies.

Microarrays themselves have known limitations due to their technical characteristics, including limited dynamic range, signal saturations, background noise, and probe cross-hybridizations. In addition, even allowing for the analysis of thousands of genes, the data collected are limited only to those genes for which probes are designed, thus leaving many unexplored. In the future, moving to other technologies (i.e., RNA-seq) would allow for the analysis of all expressed genes, thus permitting a comprehensive definition of the transcriptomic fingerprints of equine airway diseases.

Despite the aforementioned limitations, the evidence reported here suggests that innovative therapies targeting specific cellular pathways might reduce clinical symptoms in SEA-affected animals and, for MEA-affected horses, allow a quicker return to competition. Finally, the similarities found with human asthma further support severe equine asthma as a spontaneous animal model for studying this common human chronic inflammatory disease of the airways.

## Figures and Tables

**Table 1:** Cytological and bacteriological examination results of BAL fluid samples from controls and MEA- and SEA-affected horses. The percentage value of the inflammatory cells (neutrophils %, macrophages %, eosinophils %, and lymphocytes %) was obtained by performing a 200 inflammatory cell count per sample on the stained slides. Then, a further count was performed, taking into account a total of 200 cells per slide and recording only the exfoliated respiratory epithelial cells (epithelial %).

		Sex	Neutrophils (%)	Macrophages (%)	Eosinophils (%)	Lymphocytes (%)	Epithelial (%)	Mast Cells (%)
**Controls**	C1	F	0	98.6	0	1.4	0	0
	C2	F	1.5	96.8	0.7	1	0	0
	C3	M	0	99	0.3	0.7	2	0
	C4	M	0.2	99.6	0	0.2	2	0
	C5	F	2	97.1	0.6	0.3	1.3	0
	C6	F	0.2	99.6	0	0.2	0.8	0
	C7	M	0.8	91.7	6.6	0	1.3	0
	***Mean ± DS***		***0.7 ± 0.80***	***97.5 ± 2.78***	***1.2 ± 2.41***	***0.5 ± 0.51***	***1.0 ± 0.84***	**0**
**MEA**	I1	M	12.2	86.3	1	3.2	3.1	0
	I2	M	11.6	85.5	0	2.9	2.5	0
	I3	M	6.1	89.8	0	4.1	3.9	0
	I4	F	5.3	89.7	0	5	2	0
	I5	F	5.9	91.3	0	2.8	2.5	0
	I6	M	10.2	83.9	1	3.9	1.3	0
	I7	M	6.8	88.9	0	4.3	3.5	0
	I8	F	8.2	89.7	0	2.1	3.5	0
	I9	F	6.8	91.3	0	1.9	1.2	0
	I10	F	5.6	88.8	1.2	3.6	1	0
	***Mean ± DS***		***7.9 ± 2.57***	***88.3 ± 2.48***	***0.3 ± 0.52***	***3.4 ± 0.98***	***2.4 ± 1.05***	**0**
**SEA**	R1	F	81	15	1	1	2	0
	R2	M	78.5	21.5	0	0	3.7	0
	R3	F	97.4	1.7	0	0.9	0	0
	R4	M	92.8	7.2	0	0	2	0
	R5	M	86.9	12.8	0	0.3	3	0
	R6	M	56.2	38.2	3.2	3.4	0.4	0
	R7	F	82.6	17.2	0	0.2	1	0
	R8	F	98.5	1.2	0	0.3	1	0
	***Mean ± DS***		***84.2 ± 13.57***	***14.3 ± 12.04***	***0.5 ± 1.14***	***0.8 ± 1.13***	***1.6 ± 1.28***	**0**

**Table 2:** Results of GSEA comparing SEA-affected horses and controls. The table reports the 18 gene sets enriched in the SEA group and the number of genes involved in the single-gene set (Size). The nominal *p*-value (NOM *p*-value) and the false discovery rate (FDR q-value) have been reported.

Gene Set Name	Size	NOM *p*-Value	FDR q-Value
Biocarta _P53_ pathway	11	0.00	0.25
Biocarta_stress_pathway	21	0.00	0.15
Reactome_MAPK_Targets_Nuclear_Events_Mediated_By_MAP_Kinases	22	0.010	0.10
Reactome_Toll_Like_Receptor_3_Cascade	43	0.012	0.13
Biocarta _IL12_ pathway	15	0.013	0.12
Reactome _Toll_Receptor_Cascades	56	0.016	0.10
Reactome _Signaling_In_Immune_System	187	0.019	0.10
Reactome _CD28_Co_Stimulation	21	0.020	0.11
Biocarta _CD40_ pathway	14	0.024	0.10
Kegg _Cytokine_Cytokine_Receptor_Interaction	125	0.027	0.09
Kegg_MAPK_Signaling_ pathway	138	0.028	0.10
Kegg _Chemokine_Signaling_ pathway	107	0.029	0.10
Reactome _Innate_Immunity_Signaling	69	0.031	0.11
Kegg _Leukocyte_Transendothelial_Migration	69	0.032	0.12
Biocarta _Toll_ pathway	25	0.034	0.10
Biocarta _IL7_ pathway	15	0.041	0.11
Biocarta _TNFR2_ pathway	15	0.045	0.10
Asthma (Chowdhary et al. [27])	174	0.048	0.11

## Data Availability

Gene expression datasets supporting the conclusion of this article are available in the GEO (Gene Expression Omnibus) repository under accession number GSE155084.

## Supplementary Materials

The following supporting information can be downloaded at: https://www.mdpi.com/article/10.3390/ani13010004/s1, Table S1: Differentially expressed genes between SEA horses and control groups; Table S2: Differentially expressed genes between MEA horses and control groups; Table S3: Differentially expressed genes between SEA and MEA horses.
